# Protein–protein complexes can undermine ultrasensitivity-dependent biological adaptation

**DOI:** 10.1098/rsif.2022.0553

**Published:** 2023-01-04

**Authors:** C. Jeynes-Smith, R. P. Araujo

**Affiliations:** School of Mathematical Sciences, Queensland University of Technology, Brisbane, Australia

**Keywords:** chemical reaction networks, robust perfect adaptation, Michaelis–Menten, mass-action, ultrasensitivity, feedback

## Abstract

Robust perfect adaptation (RPA) is a ubiquitously observed signalling response across all scales of biological organization. A major class of network architectures that drive RPA in complex networks is the Opposer module—a feedback-regulated network into which specialized integral-computing ‘opposer node(s)’ are embedded. Although ultrasensitivity-generating chemical reactions have long been considered a possible mechanism for such adaptation-conferring opposer nodes, this hypothesis has relied on simplified Michaelian models, which neglect the presence of protein–protein complexes. Here we develop *complex-complete* models of interlinked covalent-modification cycles with embedded ultrasensitivity, explicitly capturing all molecular interactions and protein complexes. Strikingly, we demonstrate that the presence of protein–protein complexes thwarts the network’s capacity for RPA in any ‘free’ active protein form, conferring RPA capacity instead on the concentration of a larger protein pool consisting of two distinct forms of a single protein. We further show that the presence of enzyme–substrate complexes, even at comparatively low concentrations, play a crucial and previously unrecognized role in controlling the RPA response—significantly reducing the range of network inputs for which RPA can obtain, and imposing greater parametric requirements on the RPA response. These surprising results raise fundamental new questions as to the biochemical requirements for adaptation-conferring Opposer modules within complex cellular networks.

## 1. Introduction

The ability to adapt to sustained disturbances and input stimuli, and to maintain key system properties within tight tolerances, is a distinctive and ubiquitously observed feature of biological systems across all domains of life [1–8]. This crucial biological response has been studied mathematically in its idealized form, known as robust perfect adaptation (RPA), where the concentration of a particular molecule or activation state returns exactly to a prescribed baseline—its *setpoint*—after a disturbance or input to the system, for a wide range of possible network parameters (e.g. total expression levels for the interacting molecules) [2,6]. RPA has been identified as an essential signalling response in biological contexts as diverse as directed cell migration in single-celled organisms [8–22], complex sensory systems [23–28], osmoregulation in yeast and bacteria [29,30], plasma mineral homeostasis [31] and morphogen regulation and patterning during development [32,33]. Crucially, loss of RPA has been linked to a wide variety of diseases in multicellular organisms, including drug addiction, chronic pain, and cancer initiation and progression [34–39].

Importantly, RPA is a structural property of networks and is not dependent on fine-tuning of system parameters. The complete solution space for RPA-capable network designs has now been identified in full generality [2], for networks of unlimited size and complexity. In fact, it is now known that all RPA-capable networks are decomposable into modules, of which there are two broad, yet well-defined, classes: Opposer modules, and Balancer modules [2]. Opposer modules are a generalization of the three-node RPA solution known as ‘negative feedback with buffer node’ (NFB) first identified by Ma *et al.* [7] through extensive computational searching. All Opposer modules contain at least one negative feedback loop, and have at least one computational node (known as an ‘opposer node’) embedded into the feedback loop. In complex networks consisting of a large number of interacting molecules, Opposer modules may contain distributed integral controllers known as ‘Opposing Sets’, consisting of multiple interlinked negative feedback loops containing special arrangements of opposer nodes [2]. Balancer modules, by contrast, generalize the three-node ‘incoherent feed-forward loops with proportioner node’ (IFFL) identified by Ma *et al.* [7]. Balancer modules consist of an arbitrary number of parallel pathways, at least two of which must be incoherent in nature, and which have special computational nodes known as ‘balancer nodes’ embedded into them [2]. In general, RPA-capable networks can contain any number of Opposer and/or Balancer modules, connected together according to well-defined interconnectivity rules, to orchestrate highly complex RPA-capable networks [2,40,41].

**Figure 1.**
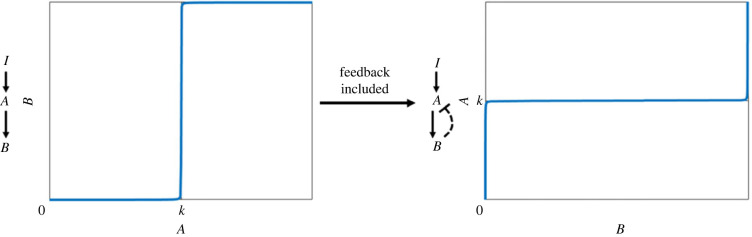
The relationship between ultrasensitivity and RPA. In an open-loop signalling cascade (left), an ultrasensitivity-generating mechanism at the level of a single protein (*B*) creates a reversible switch whereby the output can exist in either a low activity state, or a high activity state, with a vanishingly narrow transition zone (for *A* ≈ *k*) between the two states. If this ultrasensitivity-generating mechanism is embedded into a negative feedback loop (right), the narrow transition zone for the ultrasensitive switch is converted into a narrow tolerance around an RPA setpoint (*k*) for the upstream molecule *A*.

A central question in biochemical network theory remains how complex organisms incorporate these well-defined RPA-conferring network structures into their signalling networks [2,40]. Until now, most known Balancer modules have been identified in very small signalling networks such as two-component regulatory systems in bacteria [14,30,42,43], and two- or three-node transcription networks across a variety of cell types [44,45]. By contrast, RPA in large and highly complex signalling networks such as mammalian signal transduction networks is thought to depend on feedback structures, and hence, Opposer modules [2,5,40]. The essential ingredient for all Opposer modules is the embedding of at least one *opposer node*—a collection of chemical reactions which computes the integral of the deviation between the RPA setpoint and the instantaneous signalling level of an RPA molecule—into the overarching feedback structure of the module [2]. Currently, there are just two known types of opposer node—antithetic integral control [46,47] and ultrasensitivity-generating motifs [40,48]. Antithetic integral control is a universal ‘opposer node’ that confers an exact (i.e. ‘perfect’) form of RPA, which has been identified in endogenous cellular networks in the form of sigma/anti-sigma factors [42], for instance. Antithetic integral control has also been implemented successfully in a variety of synthetic bionetworks [6,13,46,47]. Ultrasensitivity-dependent opposer nodes, on the other hand, remain a mostly theoretical class of RPA-promoting signalling structures that are thought to confer an ‘almost perfect’ form of RPA [7,10,40,48–57], with evidence suggesting they play a role in the regulation of cellular signal transduction pathways [5], and bacterial chemotaxis [58–61] specific instances of this type of opposer node are yet to be identified in cells. We depict the relationship between ultrasensitivity and RPA in figure 1.

**Figure 2.**
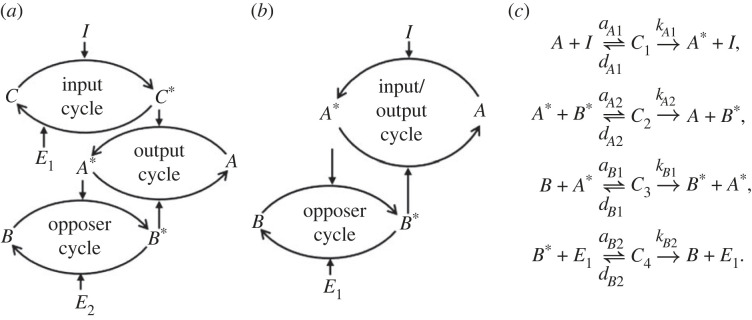
Network diagrams for (*a*) the three-node network analysed by Ma *et al.* [7] and (*b*) the corresponding reduced two-node network with a single node representing both input and output. (*c*) The graph structure for the set of chemical reactions corresponding to the network in (*b*), from which either *complex-complete* mass-action equations [48] or simplified Michaelis–Menten equations (neglecting all protein–protein complexes) can be derived. In each case, the active form of protein *X* is denoted X*. The four intermediate protein–protein complexes are denoted by *C*_1_, *C*_2_, *C*_3_ and *C*_4_. The association, dissociation and catalytic rate constants are given by *a*_*i*_, *d*_*i*_ and *k*_*i*_, respectively, as shown.

The mathematical evidence for the creation of RPA-promoting opposer nodes via ultrasensitive switches is largely linked to the landmark computational study undertaken by Ma *et al.* [7]. We depict the essential mathematical framework of the three-node signalling structures considered by Ma *et al.* [7] in figure 2*a*. In that study, the focus was specifically on three-node networks and therefore the feedback structure was predicated on the input and output nodes being distinct from one another. It was later shown [2,5,40] that two nodes are sufficient for RPA in a feedback structure (Opposer module), since input and output nodes need not be distinct, as shown in figure 2*b*. The key feature of these RPA-conferring models by Ma *et al.* [7] was the embedding of an ultrasensitive switch at the location noted as an ‘opposer cycle’ (referred to as a ‘buffer node’ in the study by Ma *et al.* [7], which was restricted to three-node networks).

Importantly, Ma *et al.* [7] drew conclusions as to the putative RPA capacity of this network under the simplifying assumption of Michaelis–Menten kinetics for each covalent-modification cycle,

1.1 $$\frac{\text{d}A^{*}}{\text{d}t}=\frac{k_{A1}I_{\mathrm{tot}}(A_{\mathrm{tot}}-A^{*})}{K_{A1}+A_{\mathrm{tot}}-A^{*}}-\frac{k_{A2}B^{*}A^{*}}{K_{A2}+A^{*}}$$

and

1.2 $$\frac{\text{d}B^{*}}{\text{d}t}=\frac{k_{B1}A^{*}(B_{\mathrm{tot}}-B^{*})}{K_{B1}+B_{\mathrm{tot}}-B^{*}}-\frac{k_{B2}E_{\mathrm{tot}}B^{*}}{K_{B2}+B^{*}},$$

and under the assumption that enzyme–substrate complex concentrations could be neglected entirely. The use of Michaelis–Menten kinetics presupposes that the concentrations of any intermediate enzyme–substrate complexes may be neglected—an assumption which is generally thought to be reasonable for a single covalent-modification cycle in isolation when the total concentrations of the interconverting enzymes (e.g. *I*_tot_, *E*_tot_) are exceedingly small in comparison with that of their substrates (*A*_tot_, *B*_tot_) [62]. In many complex signalling networks of biological interest, such as cancer signal transduction networks, enzymes and substrates typically exist at comparable concentrations [63–67], and covalent-modification cycles are highly interlinked [48].

**Figure 3.**
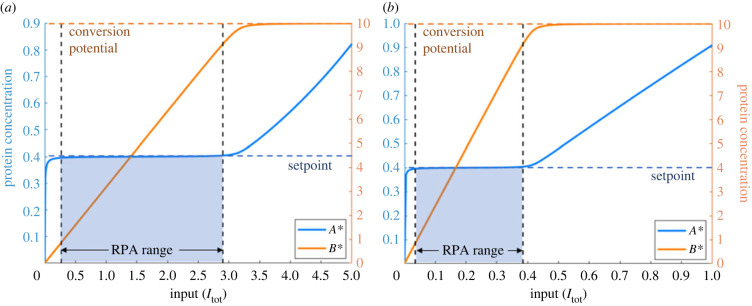
Example simulations of (*a*) the Ma *et al.* model [7] using equations (1.1) and (1.2), and (*b*) the Ferrell model [5], employing parameter sets used by Ma *et al.* and Ferrell. We highlight the range of input values over which the RPA property can be observed, i.e. the ‘RPA range’. The beginning of this range is determined by the value of input when A* is first within 1% of the estimated setpoint (dark blue dashed line), and ends when A* deviates from the setpoint by more than 1% and B* reaches the conversion potential (dark orange dashed line). Parameters: *A*_tot_ = *B*_tot_ = 10, *E*_tot_ = 1, *k*_*A*1_ = *k*_*A*2_ = 200, *k*_*B*1_ = 10, *k*_*B*2_ = 4, *K*_*A*1_ = *K*_*A*2_ = 1 and *K*_*B*1_ = *K*_*B*2_ = 0.01. Ferrell [5] equations: dA*/d*t* = *k*_*A*1_ *I*_tot_ (*A*_tot_ − A*) − *k*_*A*2_ B*A*, dB*/d*t* = *k*_*B*1_ A*(*B*_tot_ − B*)/(*K*_*B*1_ + *B*_tot_ − B* )− *k*_*B*2_ *E*_tot_ B*/(*K*_*B*2_ + B*).

Ma *et al.* [7] demonstrated analytically that RPA is dependent on the Michaelis constants in the opposer cycle (*K*_*B*1_, *K*_*B*2_) being very small in comparison with the corresponding substrate abundance (*B*_tot_ − B* and B*, respectively) (figure 3*a*). This creates a (zero-order) ultrasensitive switch in the opposer cycle [68]. The RPA setpoint, *σ*, can then be derived from equation (1.2) at steady-state, in the limit as *K*_*B*1_, *K*_*B*2_ → 0, to obtain,

$$\sigma =\frac{k_{B2}}{k_{B1}}E_{\mathrm{tot}},$$

(see the end of our Methods or Ma *et al.* [7] for the full derivation). Thus, *σ* is the basal level to which the output returns following any persistent disturbance to the network (*I*), and the location of the ultrasensitive switch in the ‘open-loop’ cascade (see figure 1).

For the sake of a complete discussion of simplified RPA frameworks, we note that Ferrell [5] later considered an even simpler version of the two-protein version of the Ma *et al.* [7] model, using simplified mass-action kinetics rather than Michaelian kinetics for the input/output cycle. We depict a characteristic output of this system in figure 3*b* using an identical parameter regime as the corresponding RPA-promoting Michaelian model (figure 3*a*). As shown in figure 3, the qualitative responses are almost identical, with a variation in the observed ‘RPA range’ being the only difference between the outputs for the two model variations. The Ferrell model [5] most closely resembles the embedding of a covalent-modification cycle into a post-transcriptionally orchestrated feedback loop, requiring de novo protein synthesis. By contrast, the Ma *et al.* model [7], involving two interlinked covalent-modification cycles, corresponds to short-term signalling within signal transduction cascades.

There also exist a number of other studies which use simple feedback structures to create RPA (and approximate RPA) capable networks, but these are also predicated on the use of Michaelis–Menten kinetics [10,49,51–53,57–59,61]. In addition, there are a range of analytical and semi-analytical methods, based on time-scale separation [43], control theoretic approaches [53,58,61] and chemical reaction network theory (CRNT) [30,69–72], which have successfully been used in the literature to identify RPA capacity (including the capacity for approximate (imperfect) RPA, as well as the special case of RPA known as absolute concentration robustness (ACR)) in a range of signalling networks. While these methods may prove useful for identifying parameter conditions that promote RPA, comparing biologically important functionalities, such as the range of inputs for which RPA obtains, elude these approaches. Additionally, frameworks which consider all intermediate chemical processes and molecules in the context of approximate RPA—as we consider here—for the simplest case of two interacting proteins, results in sufficiently complex reaction polynomials as to elude Grobner basis methods, for example.

Moreover, the pitfalls and potential inaccuracies of employing the simplified Michaelis–Menten equations for the modelling of signal processing through covalent-modification cycles are now widely appreciated [48,54,73]. Alternative quasi steady-state assumptions (QSSAs) have been successfully employed to obviate some of these difficulties in relatively simple models [54,74,75], but these approaches elude covalent-modification cycles with added regulations such as positive autoregulation (PAR) [48], or the intricate interconnection of multiple linked covalent-modification cycles. As a consequence, it is currently unknown to what extent ultrasensitivity-dependent opposer nodes could exist in biology—either endogenously within vast and complex signal transduction networks, or in a synthetic setting.

In this paper, we ask a fundamental question: has the prevalent use of simplified models given a false hope for ultrasensitivity-driven RPA mechanisms? In ‘real’ enzyme-mediated signalling networks, enzymes can only exert their activities through binding events with their substrates, creating enzyme–substrate complexes which could exist at non-negligible concentrations. Can RPA still obtain in the free active form of the output molecule, A*, when the presence of all of these protein–protein complexes are considered? Moreover, we ask a biologically crucial question which falls outside the scope of the Ma *et al.* [7] study, and which is frequently neglected in RPA studies: if RPA is indeed possible, for what range of inputs does it obtain? In biological applications, the presence of RPA for only a negligible range of inputs is not substantially different, from a functional standpoint, from not having RPA at all.

## 2. Methods

We construct here a minimal version of an ultrasensitivity-driven Opposer module, which explicitly considers all protein–protein interactions, and all intermediate molecular species—a framework we call *complex-complete* [48]. First, we consider the mass-action equations induced by the reaction scheme depicted in figure 2*c*:

2.1 $$\frac{\text{d}A}{\text{d}t}=d_{A1}C_{1}+k_{A2}C_{2}-a_{A1}AI,$$

2.2 $$\frac{\text{d}A^{*}}{\text{d}t}=d_{A2}C_{2}+k_{A1}C_{1}+d_{B1}C_{3}+k_{B1}C_{3}-a_{A2}A^{*}B^{*}-a_{B1}A^{*}B,$$

2.3 $$\frac{\text{d}B}{\text{d}t}=d_{B1}C_{3}+k_{B2}C_{4}-a_{B1}A^{*}B,$$

2.4 $$\frac{\text{d}B^{*}}{\text{d}t}=d_{A2}C_{2}+k_{A2}C_{2}+d_{B2}C_{4}+k_{B1}C_{3}-a_{A2}A^{*}B^{*}-a_{B2}B^{*}E_{1},$$

2.5 $$\frac{\text{d}I}{\text{d}t}=d_{A1}C_{1}+k_{A1}C_{1}-a_{A1}AI,$$

2.6 $$\frac{\text{d}E_{1}}{\text{d}t}=d_{B2}C_{4}+k_{B2}C_{4}-a_{B2}B^{*}E_{1},$$

2.7 $$\frac{\text{d}C_{1}}{\text{d}t}=a_{A1}AI-d_{A1}C_{1}-k_{A1}C_{1},$$

2.8 $$\frac{\text{d}C_{2}}{\text{d}t}=a_{A2}A^{*}B^{*}-d_{A2}C_{2}-k_{A2}C_{2},$$

2.9 $$\frac{\text{d}C_{3}}{\text{d}t}=a_{B1}A^{*}B-d_{B1}C_{3}-k_{B1}C_{3}$$

2.10 $$\mathrm{and}\;\;\;\;\;\frac{\text{d}C_{4}}{\text{d}t}=a_{B2}B^{*}E_{1}-d_{B2}C_{4}-k_{B2}C_{4}.$$

Taken together, these reaction rates reveal the following four mass conservation relations:

$$\begin{array}{rl} A_{\mathrm{tot}} & =A+A^{*}+C_{1}+C_{2}+C_{3},\\ B_{\mathrm{tot}} & =B+B^{*}+C_{2}+C_{3}+C_{4},\\ I_{\mathrm{tot}} & =I+C_{1} \end{array}$$

$$\;\mathrm{and}\;E_{\mathrm{tot}}=E_{1}+C_{4}.$$

In this study, we examined 3 × 10^5^ parameter sets whereby individual parameters were selected to be of the form 10^*n*^, with *n* a uniformly distributed real number on the interval [−3, 4] for the catalytic constants and Michaelis constants, and on the interval [0, 4] for total protein abundances. We computed protein steady-states by simulating the system of mass-action equations (2.1)–(2.10) using Matlab’s ODE solver, ‘ode23s’. This solver avoids timescale issues that can occur in models with parameters and variables of largely varying magnitude [76–78]. We provide all our code in an online repository (see Data accessibility statement).

For each parameter choice, we compute a steady-state dose–response profile, recognizing that RPA is characterized by two essential features: (i) The output steady-state must ‘track’ the setpoint (predicted to be *σ* = (*k*_*B*2_/*k*_*B*1_)*E*_tot_, see the end of our Methods or Ma *et al.* [7] for derivation), for a non-negligible range of inputs to the network. This range of inputs is referred to hereafter as the *RPA range*. (ii) The steady-state concentration of some *other* protein (in this case, some form of *B*) must vary as the input varies, across the RPA range. This second condition is essential to distinguishing ‘true’ RPA from any ‘trivial’ form of RPA that can occur when any protein concentration reaches its maximum possible value. We call this maximum possible value its *conversion potential* [48] (see figure 3). It is now well-established that all RPA-capable networks have at least one ‘non-RPA’ variable, which acts as an ‘actuator’ node [42].

To determine the RPA range, we identify the smallest (*I*_*S*_) and largest (*I*_*F*_) values of the network input for which RPA obtains (if at all); then *range* = *I*_*F*_ − *I*_*S*_. Thus, *I*_*S*_ is determined by identifying the smallest input value for which (i) the RPA-exhibiting variable, at steady-state, is unchanging for successive input values (within a tolerance of 2%), and is within a small tolerance (±1%) of the estimated setpoint and (ii) some other (non-RPA) variable is changing, at steady-state, by at least 1% across the same successive input values. Then, *I*_*F*_ (>*I*_*S*_) is identified as the input value for which these conditions are first violated. Note that we examined increasing these tolerances to ±2% and ±5%, which resulted in finding extra parameters (+2% and +5%) that had RPA behaviours which were less adaptive and across a larger range of inputs for both the Michaelian and complex-complete models. These extra parameter regimes maintain the same trends as the original tolerances and therefore indicate that the original tolerances are sufficient for this analysis.

For every parameter choice, we determine whether the network is capable of achieving the RPA property by identifying values for *I*_*S*_ and *I*_*F*_ , if they exist. We then partition the parameter regimes into two groups based on whether the associated network is able to achieve RPA or not. Where RPA is achieved, we further partition the parameter regimes into subgroups based on the magnitude of the RPA range.

Due to the computational demands of generating numerical solutions to this system, for the purposes of detecting their RPA capacity or otherwise, we first undertake a relatively sparse but comprehensive search of the full parameter space (3 × 10^5^ total parameter sets). After an initial analysis of the general trends in these models, we proceeded to undertake targeted searches (10^5^ parameter sets) on more focused regions of parameter space associated with the model responses of particular interest to our study (i.e. regions compatible with RPA). These additional computational searches highlight the roles of key parameter groups (Michaelis constants, catalytic constants, total protein abundances, etc.) and their influence on RPA capacity and/or of the RPA range, while holding all other parameters fixed. The trends we observe in our targeted searches were all confirmed to reflect the trends of the initial broad search.

Lastly, the setpoint, *σ*, referred to throughout this discussion was first derived by Ma *et al.* [7] from equation (1.2) at steady-state,

$$0=\frac{k_{B1}A^{*}(B_{\mathrm{tot}}-B^{*})}{K_{B1}+B_{\mathrm{tot}}-B^{*}}-\frac{k_{B2}E_{\mathrm{tot}}B^{*}}{K_{B2}+B^{*}}.$$

In the limit as *K*_*B*1_, *K*_*B*2_ → 0, the steady-state condition tends to

$$0=k_{B1}A^{*}-k_{B2}E_{\mathrm{tot}}.$$

The estimated setpoint (for the output A*), is, therefore, given by

$$\sigma =\frac{k_{B2}}{k_{B1}}E_{\mathrm{tot}}.$$

## 3. Results

We find that the RPA capacity of the complex-complete model of embedded ultrasensitivity exhibits a number of unexpected characteristics, deviating in at least three fundamentally important ways from the predictions of simplified (Michaelian) models of ultrasensitivity-driven RPA. We consider each of these surprising findings in turn below.

### 3.1. Robust perfect adaptation is *never* achieved in the ‘free’ active form of the regulated protein (A*)

In the Ma *et al.* study [7], employing Michaelis–Menten kinetics for all enzyme catalysed reactions, it was clear that the active form of the ‘output’ protein, A*, could exhibit RPA for a wide range of parameters and system inputs provided that a protein in the feedback portion of the circuit (i.e. the opposer protein, B*, in our nomenclature) exhibited ultrasensitivity (*K*_*B*1_, *K*_*B*2_ ≪ *B*_tot_). When we relax the Michaelian assumption of negligible enzyme–substrate complexes, however, and track all protein species explicitly via our complex-complete framework, we find that A*—the ‘free’ active form of the output protein—*never* tracks the estimated setpoint (*σ*), or any other non-trivial setpoint, for any choice of biochemical rate constants or protein abundances.

**Figure 4.**
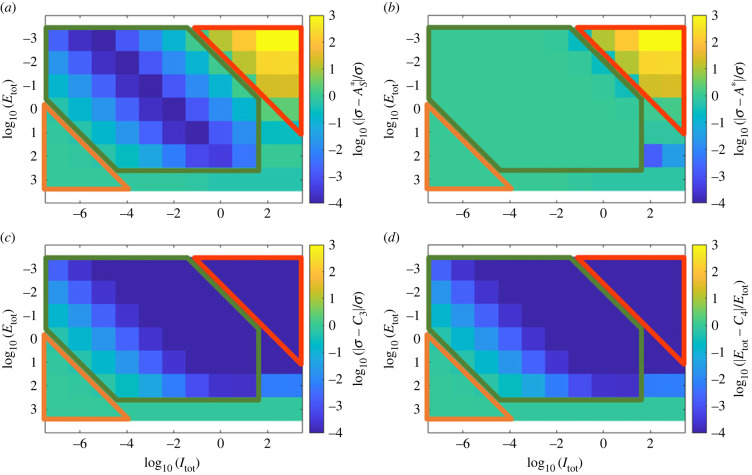
Comparison of protein concentrations for a wide range of input values (*I*_tot_) and total enzyme abundances (*E*_tot_), during three phases of the network response: prior to RPA (orange region), during RPA (green region), and after RPA has been lost (red region). We demonstrate the abundances (relative to *σ*) of: (*a*) the total input to the opposer cycle, $A_{S}^{*}$; (*b*) the free, active output protein, A*; (*c*) the complex *C*_3_ and (*d*) the complex *C*_4_ relative to the total enzyme abundance, *E*_tot_. Each heatmap represents the logged relative error of the proteins indicated in the label. As shown, RPA is associated with the conditions $A_{S}^{*}=C_{3}=\sigma$, with A* = 0, and *C*_4_ = *E*_tot_. Parameters: *A*_tot_ = *B*_tot_ = 200, *k*_*A*1_ = *k*_*A*2_ = 200, *k*_*B*1_ = 10, *k*_*B*2_ = 4, *K*_*A*1_ = *K*_*A*2_ = 1, *K*_*B*1_ = *K*_*B*2_ = 0.01, *d*_*A*1_ = *d*_*A*2_ = *d*_*B*1_ = *d*_*B*2_ = 1 and *a*_*i*_ = (*d*_*i*_ + *k*_*i*_)/*K*_*i*_.

Instead, we find that RPA can be achieved by an entirely different biochemical species. In particular, *C*_3_, being the enzyme–substrate complex consisting of the *active* form of *A* (enzyme) bound to the *inactive* form of *B* (substrate), can achieve a concentration equal to *σ*, for a significant range of inputs and for a wide variety of parameter choices. Intriguingly, under conditions where the concentration of *C*_3_ was found to track the estimated setpoint, we discovered that the concentration of A* was identically zero (or, at least, less than our chosen tolerance—see Methods). From this, we conclude that the ‘true’ RPA variable for a feedback system with embedded ultrasensitivity is the ‘total’ input to the opposer cycle which, in this case, is given by $A_{S}^{*}$ = A* + *C*_3_. Indeed, $A_{S}^{*}$ corresponds to the location of the ultrasensitive switch in the Goldbeter–Koshland model of zero-order ultrasensitivity [48,68,79]. As we show in figure 4, when $A_{S}^{*}$ tracks the value *σ* (figure 4*a*), A* = 0 (figure 4*b*). At the same time, *C*_3_ also tracks the value *σ* (figure 4*c*). In addition, *C*_4_ = *E*_tot_ (figure 4*d*), and *E*_1_ (the concentration of free enzyme) is identically zero (not shown). We recognize that these conditions correspond to the complete saturation of both interconverting enzymes for the opposer cycle—a hallmark of zero-order ultrasensitivity [68]. The Michaelian model used by Ma *et al.* [7] assumes *a priori* that all protein–protein complexes exist at negligible concentrations. Therefore, their model will never be able to identify that it is the complex, *C*_3_, which specifically tracks the setpoint once the enzymes are completely saturated, and that it is the free active molecule, A*, which is identically zero. Moreover, this result holds even in the parameter limit where the total substrate abundances (*A*_tot_, *B*_tot_) are in vast excess over the total enzyme abundance (*E*_tot_), in which the assumptions underlying the Michaelian approximation are thought to be reasonable, and the intermediate complex concentration should be negligible in principle (see figure 4 top row of each panel).

**Figure 5.**
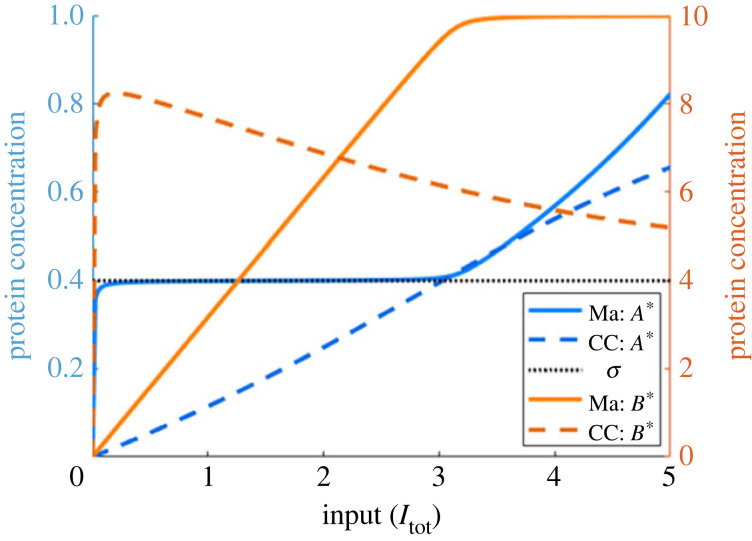
Free active protein forms in the Ma *et al.* (solid lines) [7] and complex-complete (CC) (dashed lines) models under the same parameter regime. Parameters: *A*_tot_ = *B*_tot_ = 10, *E*_tot_ = 1, *k*_*A*1_ = *k*_*A*2_ = 200, *k*_*B*1_ = 10, *k*_*B*2_ = 4, *K*_*A*1_ = *K*_*A*2_ = 1, *K*_*B*1_ = *K*_*B*2_ = 0.01, *d*_*A*1_ = *d*_*A*2_ = *d*_*B*1_ = *d*_*B*2_ = 1 and *a*_*i*_ = (*d*_*i*_ + *k*_*i*_)/*K*_*i*_.

In figure 5, we provide a representative comparison between the dose–response profile of an RPA-exhibiting Michaelian model (as used by Ma *et al.* [7]) and that of the corresponding complex-complete model. As shown, the Michaelian model bears all the hallmarks of RPA: one variable (A*) which maintains a fixed value (*σ*) over a range of system inputs, while another variable in the network (B*) varies with the input. For the corresponding complex-complete model, on the other hand, A* increases monotonically with the system input, for all inputs; by contrast, B* initially increases steeply with the input, then exhibits a prozone effect, whereby the output gradually decreases after reaching some peak abundance. (We refer the interested reader to Jeynes-Smith & Araujo [48] for other examples of covalent-modification cycles that exhibit a prozone effect of this type).

**Figure 6.**
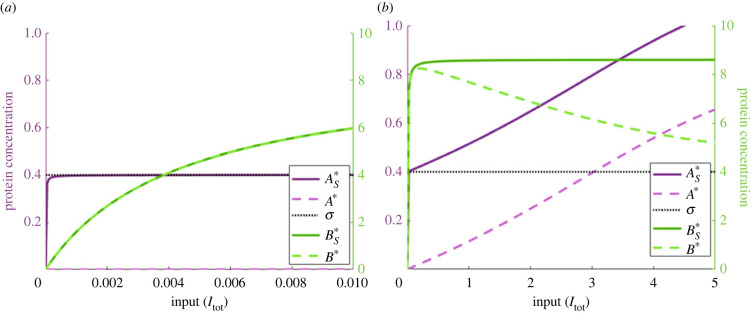
Comparison of the free active proteins (dashed lines) and the corresponding total active protein pools (comprising free protein plus complex with downstream substrate, solid lines) in the complex-complete model, for two input ranges: (*a*) *I*_tot_ ∈ [0, 10^−2^] and (*b*) *I*_tot_ ∈ [0, 5] (as seen in figure 5). The total active protein pools are given by: $A_{S}^{*}=A^{*}+C_{3}$ and $B_{S}^{*}=B^{*}+C_{2}$. In (*a*), we restrict the input domain in order to demonstrate how the complex-complete network is capable of achieving RPA, while (*b*) demonstrates the deviation of the output from the estimated setpoint. Parameters: *A*_tot_ = *B*_tot_ = 10, *E*_tot_ = 1, *k*_*A*1_ = *k*_*A*2_ = 200, *k*_*B*1_ = 10, *k*_*B*2_ = 4, *K*_*A*1_ = *K*_*A*2_ = 1, *K*_*B*1_ = *K*_*B*2_ = 0.01, *d*_*A*1_ = *d*_*A*2_ = *d*_*B*1_ = *d*_*B*2_ = 1 and *a*_*i*_ = (*d*_*i*_ + *k*_*i*_)/*K*_*i*_.

When $A_{S}^{*}$ is considered, on the other hand, as depicted in figure 6, we find that RPA is achieved, with $A_{S}^{*}\approx \sigma$ (and A* = 0), albeit for a very small range of inputs (figure 6*a*). As the input to the system is increased by several orders of magnitude, however, $A_{S}^{*}$ readily exceeds the specified tolerance around the estimated setpoint and increases monotonically with the system input. Strikingly, we see that RPA is lost precisely when A*, the putative RPA variable in the Michaelian approximation of the system’s rate equations, acquires a non-zero value: the concentration of the complex *C*_3_ continues to track a value of *σ*, (figure 6*b*)—an instance of ‘trivial’ RPA, since the output to the opposer cycle, $B_{S}^{*}=B^{*}+C_{2}$ has reached its maximum value and no longer varies with the system input (figure 6*b*).

### 3.2. Previously unrecognized constraints on protein abundances to support robust perfect adaptation range

In our extensive simulations of the two versions of the two-protein pathway, the Michaelian model often correctly identifies the occurence of RPA, but consistently overestimates the range of inputs over which it obtains. When comparing the two models under the same parameter regimes, we observed that, for the Michaelian model, approximately 6.4% of the sampled parameter space supported RPA. By contrast, in the complex-complete model, approximately 5.5% of the parameter space supported RPA; about 3.8% of the parameter space was compatible with RPA for both models. To attempt to explain the discrepancies in RPA-compatible parameter regimes for the two different models, we closely scrutinized the role of total protein abundances, considering the fact that Michaelis–Menten kinetics should in principle be valid when *A*_tot_, *B*_tot_ ≫ *E*_tot_.

**Figure 7.**
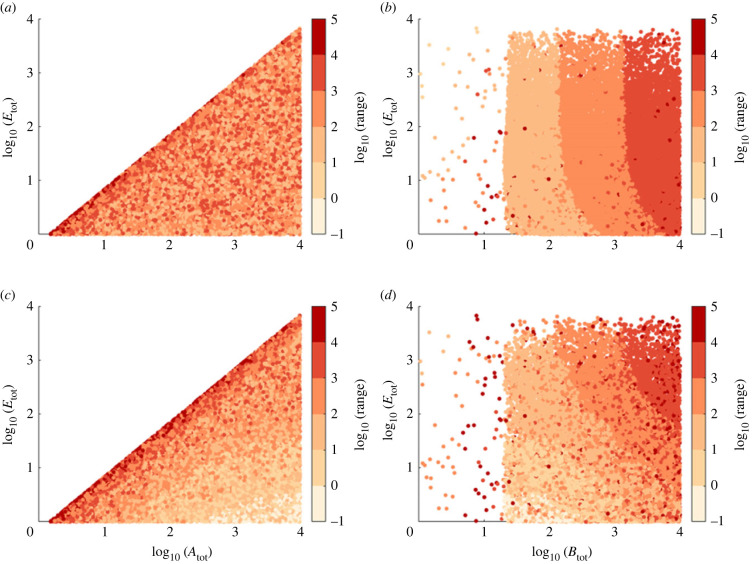
The influence of total substrate and enzyme abundances on RPA capacity in the Michaelian (Ma *et al.* [7]) model for two sensitivity regimes in the input/output cycle: (*a*,*b*) low sensitivity (*K*_*A*1_ = 700, *K*_*A*2_ = 500) and (*c*,*d*) high sensitivity (*K*_*A*1_ = 7, *K*_*A*2_ = 5). When RPA obtains, the RPA range is indicated by colour. As shown, the Michaelian model of RPA imposes strict constraints on *A*_tot_ relative to *E*_tot_ (*a*,*c*), with no constraints on *B*_tot_ (*b*,*d*). However, *B*_tot_ exerts a significant influence on the RPA range. Parameters: *k*_*A*1_ = 7, *k*_*A*2_ = 5, *k*_*B*1_ = 2, *k*_*B*2_ = 3, *K*_*B*1_ = 2 × 10^−2^ and *K*_*B*2_ = 3 × 10^−2^.

In figure 7, we show that for the Michaelian simplification, RPA can only obtain (in the variable A*) if *A*_tot_ > (*k*_*B*2_/*k*_*B*1_)*E*_tot_ (figure 7*a*,*c*), while no such constraint exists for the total abundance of the opposer protein (*B*_tot_) (figure 7*b*,*d*). Nevertheless, we discovered that *B*_tot_ exerts a strong influence on the RPA range, independently of the value of *E*_tot_, once *E*_tot_ exceeds a threshold value determined by the sensitivity of the input/output cycle (governed by the values of *K*_*A*1_ and *K*_*A*2_) (see figure 7*b*,*d*).

**Figure 8.**
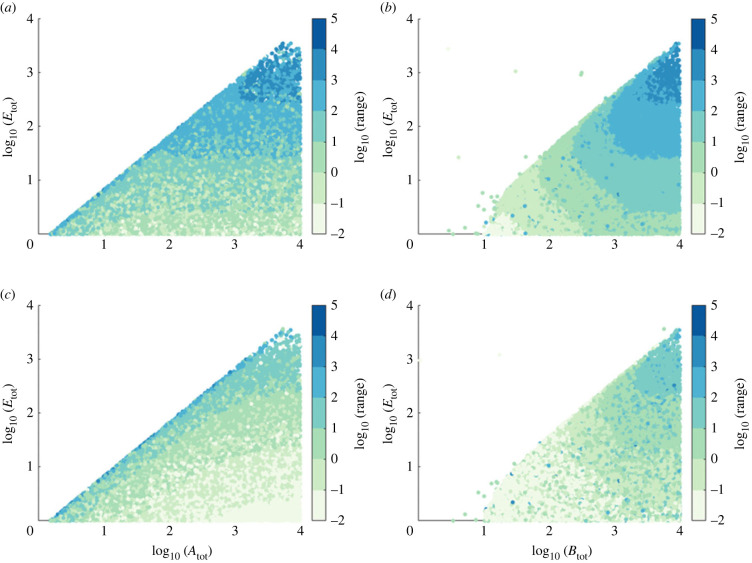
The influence of total substrate and enzyme abundances on RPA capacity in the complex-complete model for two sensitivity regimes in the input/output cycle: (*a*,*b*) low sensitivity (*K*_*A*1_ = 700, *K*_*A*2_ = 500) and (*c*,*d*) high sensitivity (*K*_*A*1_ = 7, *K*_*A*2_ = 5). When RPA obtains, the RPA range is indicated by colour. As shown, the complex-complete model of RPA imposes strict constraints on all total protein abundances, all of which can also affect the RPA range. Parameters: *k*_*A*1_ = 7, *k*_*A*2_ = 5, *k*_*B*1_ = 2, *k*_*B*2_ = 3, *K*_*A*1_ = 7, *K*_*A*2_ = 5, and *K*_*B*1_ = 2 × 10^−2^, *K*_*B*2_ = 3 × 10^−2^, *d*_*A*1_ = *d*_*A*2_ = *d*_*B*1_ = *d*_*B*2_ = 1 and *a*_*i*_ = (*d*_*i*_ + *k*_*i*_)/*K*_*i*_.

For the complex-complete model, on the other hand, we find that while the constraint *A*_tot_ > (*k*_*B*2_/*k*_*B*1_)*E*_tot_ still holds (figure 8*a*,*c*), the capacity for RPA also strictly requires *B*_tot_ > ((*k*_*B*2_/*k*_*B*1_) + 1)*E*_tot_ (figure 8*b*,*d*). Moreover, both *B*_tot_ and *E*_tot_ exert a strong influence on RPA range (figure 8*b*,*d*).

That the condition *A*_tot_ > (*k*_*B*2_/*k*_*B*1_)*E*_tot_ = *σ* must be satisfied for RPA to obtain is self-evident, since the total abundance of the protein *A* must be at least as great as the setpoint. This condition must, therefore, hold for *any* model of RPA, however approximate or simplified. But when the presence of protein–protein complexes are taken into account, the protein abundance requirements of the network are more subtle. Indeed, an analysis of the system’s steady-states, taking into consideration its mass conservation relations, makes clear that the presence of enzyme–substrate complexes imposes a number of additional constraints on the total protein abundances required for RPA.

Recall that *B*_tot_ = *B* + B* + *C*_2_ + *C*_3_ + *C*_4_ (see system equations given in Methods). We showed in §3.1 that when RPA obtains, *C*_3_ = *σ* = (*k*_*B*2_/*k*_*B*1_)*E*_tot_ and A* = 0, with *C*_4_ = *E*_tot_ and *E*_1_ = 0. Under these conditions, the maximum value of $B_{S}^{*}=B^{*}+C_{2}$ occurs when *B* = 0, giving

3.1 $$B_{S}^{*}=B_{\mathrm{tot}}-E_{\mathrm{tot}}\left(1+\frac{k_{B2}}{k_{B1}}\right).$$

**Figure 9.**
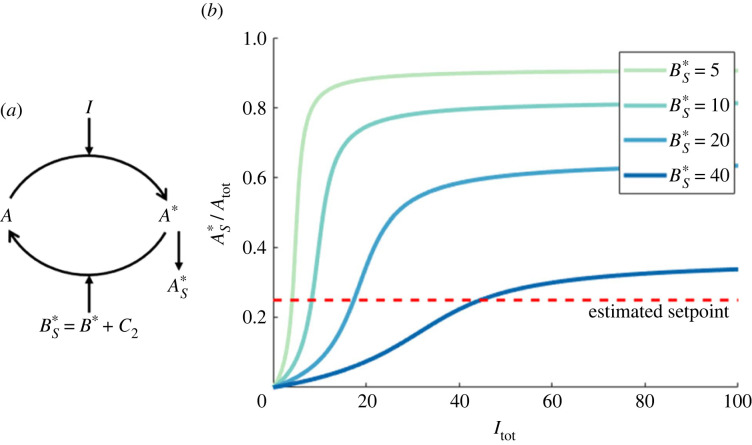
(*a*) The input/output cycle, considered as a single reversible covalent-modification cycle. (*b*) The dose response profile for this cycle, for varying enzyme abundances, $B_{S}^{*}$. As shown, increasing $B_{S}^{*}$ increases the amount of input (*I*_tot_) required to generate a particular output value for $A_{S}^{*}$. When considering the maximum possible value that $B_{S}^{*}$ can take (as determined by the opposer cycle in the full system), the intersection of a curve with the estimated setpoint line indicates the maximum value of *I*_tot_ for which RPA obtains. If the maximum possible value of $B_{S}^{*}$ is reduced (lighter curves), the maximum possible value of *I*_tot_ for which RPA obtains is reduced commensurately, thereby reducing the RPA range. Parameters: *A*_tot_ = 100, *k*_*A*1_ = *k*_*A*2_ = *k*_*B*1_ = *k*_*B*2_ = 1, *K*_*A*1_ = *K*_*A*2_ = 0.01, *d*_*A*1_ = *d*_*A*2_ = 1 and *a*_*i*_ = (*d*_*i*_ + *k*_*i*_)/*K*_*i*_.

Now, considering the input/output cycle in isolation, detached from the opposer cycle (figure 9*a*), the output for that cycle, $A_{S}^{*}$, approaches its half-maximal value as $I_{\mathrm{tot}}\to (k_{A2}/k_{A1})B_{S}^{*}$ (see [48,79] for further details on this point). Therefore, if the maximum possible $B_{S}^{*}$ for the system is increased, a greater range of values for *I*_tot_ are compatible with achieving the RPA setpoint ($A_{S}^{*}=\sigma =(k_{B2}/k_{B1})E_{\mathrm{tot}}$) (see figure 9*b*). Thus, for a given value of *E*_tot_, increasing *B*_tot_ increases the RPA range. Moreover, equation (3.1) makes clear that the capacity for RPA strictly requires *B*_tot_ > *E*_tot_(1 + (*k*_*B*2_/*k*_*B*1_)).

In addition, since *E*_tot_ determines the setpoint, which is tracked by the variable $A_{S}^{*}$ under RPA-permissive conditions, it is clear from the structure of the input/output cycle (see figure 9*a*) that, provided the minimum requirements for *A*_tot_ and *B*_tot_ are met, a greater range of *I*_tot_ values are compatible with RPA for a larger value of *E*_tot_ (with a proportionally larger value of $A_{S}^{*}$) (see figure 9*b*), thereby allowing a larger RPA range.

**Figure 10.**
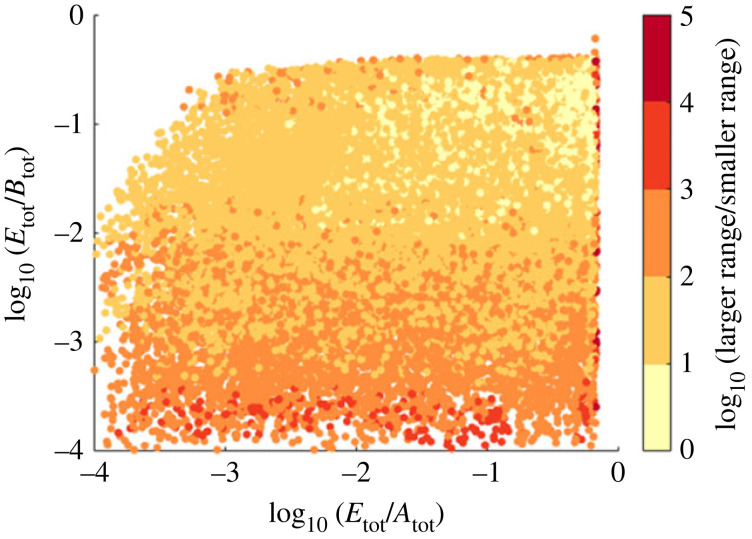
Increasing *E*_tot_ relative to *A*_tot_ and *B*_tot_ in both the Ma *et al.* [7] and complex-complete models results in the convergence of the RPA ranges across the two model types. For each parameter choice, we compare the RPA ranges predicted by the two different models, in each case dividing the larger range by the smaller range. Note that the RPA range for the Michaelian model is always the larger of the two. The log of this result is thus greater than zero, and the closer to zero (light yellow) the result becomes, the more similar are the RPA ranges for the two models. Parameters shown here: *k*_*A*1_ = 7, *k*_*A*2_ = 5, *k*_*B*1_ = 2, *k*_*B*2_ = 3, *K*_*A*1_ = 7, *K*_*A*2_ = 5, *K*_*B*1_ = 2 × 10^−2^, *K*_*B*2_ = 3 × 10^−2^, *E*_tot_, *A*_tot_, *B*_tot_ ∈ [1, 10^4^], *d*_*A*1_ = *d*_*A*2_ = *d*_*B*1_ = *d*_*B*2_ = 1 and *a*_*i*_ = (*d*_*i*_ + *k*_*i*_)/*K*_*i*_.

Most significantly, our extensive computational searches, taken together, highlight the fact that the Michaelian model of ultrasensitivity-driven RPA characteristically overestimates the RPA range, in comparison with the ‘true’ RPA range calculated by the complex-complete framework. In fact, although the assumption of negligible enzyme–substrate complexes (central to the Michaelian simplification) requires *A*_tot_, *B*_tot_ ≫ *E*_tot_, i.e. substrate concentrations exist in vast excess over enzyme concentrations, we found that the greatest agreement between the two modelling frameworks occurred when *A*_tot_ ≈ *B*_tot_ ≈ *E*_tot_. Furthermore, the more the total protein concentrations *A*_tot_, *B*_tot_ exceed the enzyme concentration *E*_tot_, the more the Michaelian model overestimates the RPA range (figure 10). Paradoxically, then, under the very conditions that might seem to justify the use of the Michaelis–Menten equation, this simplified model is most misleading in its predictions of the system’s RPA capacity.

### 3.3. Robust perfect adaptation imposes parametric constraints on both opposer and input/output cycles

In their simplified (Michaelian) model of RPA, neglecting the presence of protein–protein complexes, Ma *et al.* [7] identified a very specific parameter requirement for RPA—namely, very small Michaelis constants in the opposer cycle relative to the total abundance of the opposer protein. As noted earlier, this specific parametric condition engenders zero-order ultrasensitivity in the opposer cycle. Moreover, Ma *et al.* [7] showed analytically that, in the Michaelian framework, the presence of RPA was unaffected by any other parameters in the network.

**Figure 11.**
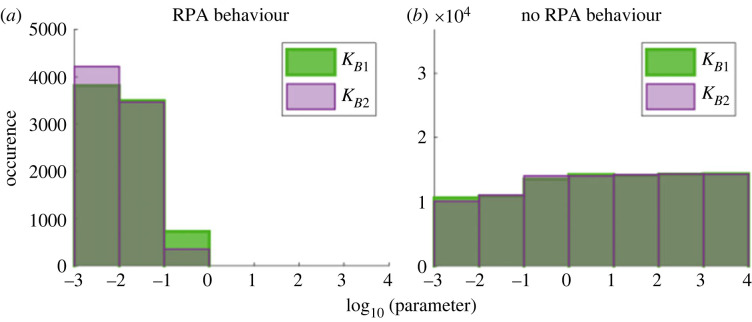
Histograms of (*a*) RPA versus (*b*) non-RPA responses for varying Michaelis constants in the opposer cycle. Small Michaelis constants are a necessary, but insufficient, condition for generating the RPA response. One hundred thousand simulations were run with Michaelis constants being randomly selected from 10^−3^ to 10^4^. Parameters: *A*_tot_ = *B*_tot_ = 10, *E*_tot_ = 1, *k*_*A*1_ = *k*_*A*2_ = *k*_*B*1_ = *k*_*B*2_ = 1, *d*_*A*1_ = *d*_*A*2_ = *d*_*B*1_ = *d*_*B*2_ = 1 and *a*_*i*_ = (*d*_*i*_ + *k*_*i*_)/*K*_*i*_.

By contrast, once all protein–protein interactions and intermediate complexes are included, we find that RPA imposes tight parameter constraints on *both* the opposer cycle *and* the input/output cycle. We confirm that RPA requires small Michaelis constants in the opposer cycle, *K*_*B*1_ and *K*_*B*2_ (figure 11*a*), and that as these Michaelis constants increase (figure 11*b*), the RPA property is lost. However, we also identified a significant region of our parameter space with very small values of *K*_*B*1_ and *K*_*B*2_, for which RPA did not obtain. On closer examination of parameter sets associated with RPA, we found that the Michaelis constants, *K*_*A*1_ and *K*_*A*2_, as well as the catalytic constants, *k*_*A*1_ and *k*_*A*2_, for the input/output cycle, had a significant impact on the ability of the network to exhibit RPA and on the RPA range.

**Figure 12.**
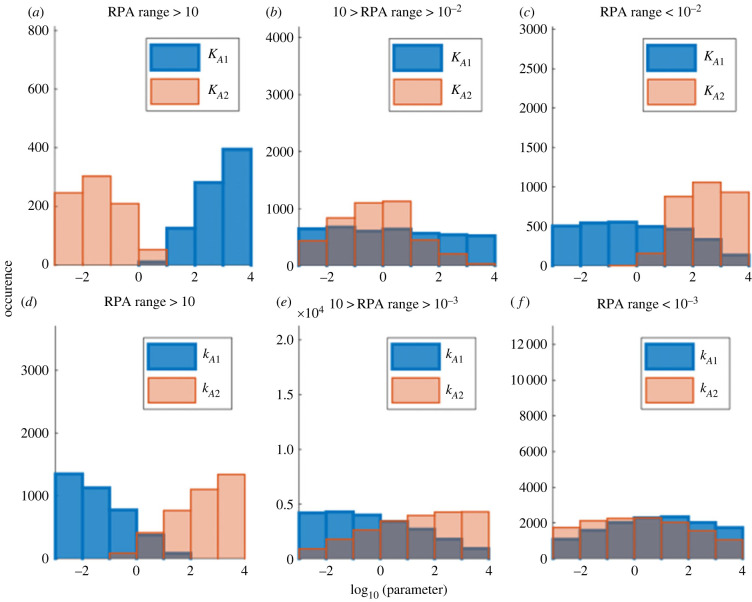
Histograms of the input/output cycle Michaelis constants (*a*–*c*), and catalytic constants (*d*–*f*), which were associated with RPA, grouped by the magnitude of the RPA range. As shown, smaller values of *K*_*A*2_ and *k*_*A*1_, and larger values of *K*_*A*1_ and *k*_*A*2_ are associated with larger RPA ranges. One hundred thousand simulations were run with Michaelis constants or catalytic constants being randomly selected from 10^−3^ to 10^4^. Parameters: *A*_tot_ = *B*_tot_ = 10, *E*_tot_ = 1, *K*_*A*1_ = *K*_*A*2_ = 1 (except where noted otherwise), *K*_*B*1_ = *K*_*B*2_ = 0.01, *k*_*A*1_ = *k*_*A*2_ = *k*_*B*1_ = *k*_*B*2_ = 1 (except where noted otherwise), *d*_*A*1_ = *d*_*A*2_ = *d*_*B*1_ = *d*_*B*2_ = 1 and *a*_*i*_ = (*d*_*i*_ + *k*_*i*_)/*K*_*i*_.

Indeed, we found that RPA in the complex-complete framework requires large values of *K*_*A*1_, and small values of *K*_*A*2_. As we show in figure 12*a*–*c*, decreasing the value of *K*_*A*1_ and increasing the value of *K*_*A*2_ reduces the RPA range. Likewise, RPA is promoted by small values of *k*_*A*1_ and large values of *k*_*A*2_, with a marked reduction in RPA range resulting from increasing values of *k*_*A*1_ and reducing values of *k*_*A*2_ (figure 12*d*–*f*).

**Figure 13.**
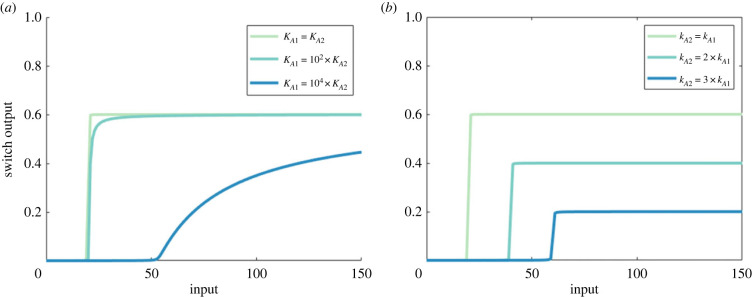
The role of (*a*) Michaelis constants and (*b*) catalytic constants in a single reversible covalent-modification cycle. Parameters: *A*_tot_ = 100, *E*_tot_ = 20, *d*_*A*1_ = *d*_*A*2_ = 1 and *a*_*i*_ = (*d*_*i*_ + *k*_*i*_)/*K*_*i*_, (*a*) *k*_*A*1_ = *k*_*A*2_ = 1, *K*_*A*2_ = 0.01, *K*_*A*1_ ∈ {0.01, 1, 100} (light to dark curves), (*b*) *K*_*A*1_ = *K*_*A*2_ = 0.01, *k*_*A*2_ = 1, *k*_*A*2_ ∈ {1, 2, 3} (light to dark curves).

The role and significance of these parameters may be elucidated by examining the input/output cycle as a single reversible covalent-modification cycle, as previously implemented in figure 9. Applying either of the parameter conditions which increase the RPA range, for the Michaelis (figure 13*a*) or the catalytic constants (figure 13*b*), results in dose–response profiles which require more input to achieve equivalent output concentrations. Importantly, the dose–responses in figure 13 are underpinned by increased protein–protein complex concentrations, and cannot be obtained by a simplified Michaelian framework, as used by Ma *et al.* [7].

## 4. Discussion and concluding remarks

Ma *et al.* [7] identified the earliest known instances of what are now known as Opposer modules and Balancer modules, generalizations of which were later shown definitively to be topological basis modules for all possible RPA-capable networks of any size and complexity [2]. Both classes of RPA basis module exhibit strict structural requirements (involving an overarching feedback structure in the case of Opposer modules, and a feed-forward structure in the case of Balancer modules) and, crucially, execute their RPA-conferring computational functions through the embedding of certain types of computational ‘nodes’ into these well-defined structures—opposer nodes, embedded into the feedback component(s) of Opposer modules; and balancer nodes, embedded into the feed-forward component(s) of Balancer modules.

Unlike Balancer nodes, which are relatively easy to construct [42], opposer nodes are now acknowledged to be notoriously difficult to create at the level of intermolecular interactions, in the form of chemical reaction networks (CRNs) [40,50,55,56]. The Ma *et al.* study [7] used the commonly employed simplification of Michaelis–Menten kinetics to model three-node networks of interlinked covalent-modification cycles, and used this modelling approximation to suggest that an ultrasensitivity-generating mechanism suffices as an opposer node (or a ‘buffer node’ in the language of that early study). As a consequence, it has been widely accepted that RPA can readily be orchestrated in signalling networks constructed from collections of enzyme-mediated post-translational modifications.

Here, we show that, when the detailed intermolecular interactions required for enzyme-mediated reactions are fully accounted for, RPA is much more difficult to achieve via an ultrasensitivity-generating mechanism than previously thought. In fact, RPA cannot be achieved, under any parametric conditions, in the ‘free’ activated form of any protein—a functionally crucial result that has been overlooked in all previous studies of RPA. Strikingly, the *only* species that can possibly achieve RPA for a non-negligible range of disturbances to the network is the ‘total’ active pool of each protein that resides, topologically speaking, between the opposer node (which exhibits ultrasensitivity) and the ‘connector node’ of the Opposer module (see [2,40] for a comprehensive overview of Opposer module topologies). This total active protein pool comprises both the free form of the activated protein, as well as a complexed form with its downstream protein substrate. In the case of the protein which directly regulates the opposer node (the latter often being referred to in the language of control theory as the *sensor* node), this total pool of active protein comprises *only* the enzyme–substrate complex, with zero concentration in the free form. This particular protein thereby exhibits RPA in the concentration of a protein–protein complex that is neglected entirely in the Michaelian framework. Moreover, this result holds even under the conditions for which the Michaelis–Menten framework should be valid (protein substrate concentrations in vast excess of enzyme concentrations).

**Figure 14.**
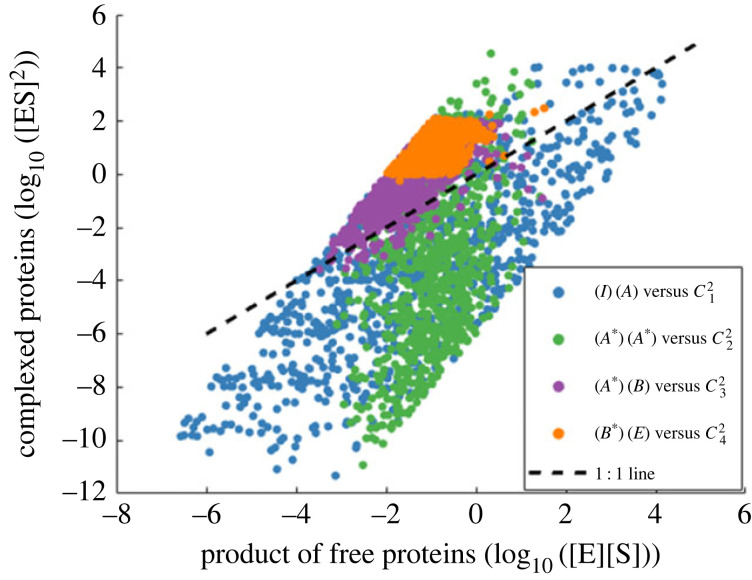
A comparison of the product of free enzyme and free substrate concentrations ([*E*][*S*]), with the square of their corresponding complex concentration ([*ES*]^2^). The tQSSA assumption employed by Ma *et al.* [7] in their simplified mass action analysis requires that for any enzyme–substrate interaction, [*ES*]^2^ ≪ [*E*][*S*]. As shown, this condition never holds for the interactions between A* and *B* (with corresponding complex *C*_3_), or between B* with *E* (with corresponding complex *C*_4_), and only rarely holds for the other enzyme–substrate interactions of the models (involving complexes *C*_1_ and *C*_2_). Each point shown above corresponds to a steady-state concentration obtained from model simulations using the parameter sampling discussed in our Methods section.

We acknowledge that Ma *et al.* [7], in their electronic supplementary material, make use of a total quasi steady-state assumption (tQSSA) to arrive at a simplified set of dynamical mass-action equations that correspond to the Michaelis–Menten versions featured throughout their main paper. These simplifications support our finding here that a certain ‘total enzyme’, equivalent to $A_{S}^{*}=A^{*}+C_{3}$ (in the nomenclature of our study), can achieve RPA. But importantly, the tQSSA employed in that analysis assumes that, for each of the interacting ‘nodes’, the square of the complex concentration is always much smaller than the product of the corresponding free enzyme and free substrate concentrations (i.e. [*ES*]^2^ ≪ [*E*][*S*] for all reactions). As we show in figure 14, this condition on complex concentrations is never achieved for complex concentrations *C*_3_ (the RPA variable) or *C*_4_, and is only rarely achieved for the other complex concentrations in the model, sampled over the entirety of our parameter space. In addition, this simplified analysis by Ma *et al.* fails to make the crucial observation that when RPA obtains at $A_{S}^{*}$, A* = 0 and $C_{3}=A_{S}^{*}=\sigma$—a result of crucial functional significance, as we discuss further in the paragraphs to follow.

For our model, being a minimal Opposer module, the sensor protein (*A*) and the opposer protein (*B*) are the only two proteins considered, with the sensor protein also playing the additional role of input/output node for the module. It is clear from the definitive topological properties of Opposer modules that any additional proteins between the sensor and the connector nodes (see [2]) will also exhibit RPA; from our complex-complete model reported here, it follows that each of these proteins will exhibit RPA in the total pool of the activated form, but will generally comprise a non-zero concentration of the free form since these proteins regulate a *non-ultrasensitive* downstream protein [48]. Indeed, the partition of the RPA-exhibiting total pool into free and complexed forms depends on the sensitivity of the protein immediately downstream, and will, therefore, be highly variable [48].

In any case, since RPA can only obtain in a protein pool that comprises both a free and a complexed form of a protein, the biological usefulness of this form of RPA is dependent upon the ability of such a protein to orchestrate its downstream extramodular functions equally in either form. In other words, the conformation of an RPA-exhibiting protein must allow it to bind its downstream target(s) with equal affinity in either its free or complexed (with its intramodular substrate) form. Goldbeter & Koshland [68], for example, discuss the possibility of an output protein that is an enzyme such as phosphorylase, where its active site is free in the protein complexes, so that the complexed form may be just as active as the free form. Undoubtedly, the possibility of exerting the same enzymatic activity in both free and complexed forms will vary from protein to protein, and is a property that could be studied through *in vitro* assays. Should ultrasensitivity-dependent RPA actually be implemented by complex cellular networks, this necessary structural property of RPA-capable proteins may provide valuable clues as to the portion of the proteome that could participate in such RPA-conferring networks.

Beyond the identity of the RPA-capable network variable, our complex-complete modelling framework also highlights the fact that RPA places much greater constraints on the feasible parameter space than suggested by the Michaelian framework [7]. In addition, our work also considers the important concept of ‘RPA range’—that is, the range of network disturbances or stimuli for which RPA obtains—which has largely been overlooked in previous studies (including the simplified mass-action analysis undertaken by Ma *et al.* [7]), which have focused purely on the presence or absence of RPA. This is a crucially important property, since networks with only a vanishingly small RPA range may provide no functional benefits, in practice, in comparison with networks that cannot exhibit RPA at all. In this connection, it is intriguing to observe that parameter alterations that increase RPA range could ultimately jeopardize the very presence of RPA. For instance, we have shown that decreasing the abundances of protein substrates (e.g. *A*_tot_) tends to increase the RPA range; but we also show that RPA is only possible for protein abundances above a certain threshold (i.e. *A*_tot_ ≥ (*k*_*B*2_/*k*_*B*1_)*E*_tot_). In a population of cells, where protein abundances are inevitably highly variable, it may thus be advantageous to the robustness of signalling functions (e.g. via RPA capacity) across the entire population to limit the total expression levels of proteins to maintain enzymes and substrates at comparable concentrations; on the other hand, this strategy risks the loss of robustness altogether in a subset of the population should substrate abundances in those cells fall below the threshold level.

Importantly, our study highlights the fact that Michaelian models characteristically overestimate the RPA range. In fact, the very conditions which are generally thought to justify the use of the Michaelian simplification (protein substrate concentrations, e.g. *A*_tot_, *B*_tot_, in vast excess over enzyme concentrations, e.g. *E*_tot_), result in the largest discrepancy in RPA range in comparison with the complex-complete framework.

Ma *et al.* [7], in neglecting the possibility of significant enzyme–substrate complexes from the outset, ignore the subtleties that occur (with respect to enzyme saturation) in the partitioning of each protein into such a significant number of free and complexed forms. Indeed, there are a number of complexed forms for each of the two proteins, owing to the fact that both *A* and *B*, in their various possible forms, can act both as enzymes and substrates. The Ma *et al.* [7] framework characteristically overestimates the RPA range, as we demonstrate in §3.2. When the total abundances *A*_tot_ and *B*_tot_ are reduced to be a similar order of magnitude to the ‘exogenous enzyme’, *E*_1_, which determines the RPA setpoint, and the opposer cycle is at enzyme saturation (*K*_*B*1_, *K*_*B*2_ ≪ 0) and the input/output cycle far from saturation [7,68], this leaves virtually no opportunity for additional enzyme–substrate complexes in the input/output cycle (since, under these conditions, *A*_tot_ is almost completely assigned to the form A*B = *C*3 = (*k*_*B*2_/*k*_*B*1_)*E*_tot_. The only opportunity to have enzyme–substrate complexes in the input/output cycle (*IA* and B*A*) comes from whatever remains once *A*_tot_ has been distributed to A*B = (*k*_*B*2_/*k*_*B*1_)*E*_tot_, which must always hold whenever RPA obtains. Thus, when total abundances of proteins are restricted in this manner, the paradoxical lack of enzyme–substrate complexes in the input/output cycle (in contrast to the opposer cycle, where enzymes are saturated completely) thus best reflects the expectation of low complexes assumed in the Michaelian model, and the RPA range is increased (to be comparable to the relatively large RPA range in the Michaelian models).

The shortcomings of the Michaelis–Menten equation to accurately capture the processing of biochemical signals via enzyme-mediated signalling events are now widely acknowledged, particularly in contexts involving highly intricate intermolecular interactions, and this study adds to the growing body of literature that cautions against its indiscriminate use [48,54,63–67,73,80,81]. Michaelian models of RPA such as those developed by Ma *et al.* [7] assume *a priori* that protein–protein complexes exist at sufficiently small concentrations that they can be neglected. By contrast, the present study makes clear that the existence of protein–protein complexes is key to the very existence of RPA via ultrasensitivity-generating mechanisms, and *cannot* be neglected. The centrally important role of protein–protein complexes (e.g. in the form of sigma/anti-sigma factor complexes) in antithetic integral control—the other type of opposer mechanism, distinct from the ultrasensitivity-dependent mechanism considered here—is already well-established [6]. Moreover, antithetic integral control has been demonstrated to achieve RPA in stochastic frameworks, even when unstable steady-state is predicted in the corresponding deterministic framework. By contrast, the present study has only focused on deterministic simulations of ultrasensitivity-dependent adaptation, and it is likely that introducing stochastic noise and intracellular spacial variation will only further limit the biological applicability of these ultrasensitivity-dependent mechanisms [56]. Since RPA is a ubiquitously observed and functionally crucial network response for biologic systems across all domains of life, the problem of constructing biologically useful opposer nodes through intermolecular interactions, particularly in the context of vast and highly complex signal transduction networks where Opposer modules are considered most prevalent, is vitally important to the evolution of life.

## Data Availability

All code used in this study is available at: https://github.com/JeynesSmith/JeynesSmithPerfectAdaptation2022.
